# Establishment and Validation of a Cell-Based Relative Potency Method for Respiratory Syncytial Virus mRNA Vaccine Drug Substance

**DOI:** 10.3390/vaccines14050401

**Published:** 2026-04-29

**Authors:** Shifeng Zheng, Xiaoqin Zhang, Wenhua Li, Hui Zhao

**Affiliations:** 1 Shenzhen Shenxin Biotechnology Co., Ltd., Shenzhen 518000, China; zhangxiaoqin@innorna.com (X.Z.); liwenhua@innorna.com (W.L.); 2 National Institutes for Food and Drug Control, Beijing 102629, China

**Keywords:** respiratory syncytial virus, mRNA drug substance, in vitro assay, development and validation, potency, critical quality attributes (CQAs)

## Abstract

**Background:** An accurate, sensitive, and robust potency assay is essential for the quality control of mRNA drug substances, which are characterized by complex manufacturing processes, intricate molecular structures, and high susceptibility to degradation. Currently, mRNA vaccine manufacturers use a variety of biological potency assays, often without systematic method development or rigorous evaluation. As a result, these assays may lack sufficient accuracy and robustness, making it difficult to reliably distinguish mRNA drug substance samples with different potency levels. Therefore, there is a need for a standardized, robust, and reliable potency assay for the evaluation of mRNA drug substance samples across a range of potencies. **Methods:** In this study, we developed a cell-based relative potency assay for a respiratory syncytial virus (RSV) mRNA drug substance encoding an engineered prefusion (PreF) form of the RSV type A (RSV-A) F protein, a recognized target for RSV vaccine development. The RSV mRNA drug substance was complexed with transfection reagents and introduced into cells in vitro to enable expression of the RSV-A PreF protein, which was then quantified using a double-antibody sandwich ELISA. **Results:** Systematic optimization showed that cell line, cell density, transfection reagent, mRNA-to-transfection reagent ratios, and transfection duration all influenced assay performance. Under optimized conditions, the assay demonstrated acceptable accuracy and precision, with relative bias values ranging from −25% to 13% across the potency range of 44~156%, measured-to-expected ratios within 0.8~1.2, and relative standard deviations of 18% and 16% for intra- and inter-assay precision, respectively. Furthermore, the optimized potency assay effectively distinguished mRNA drug substance samples with varying potency levels. **Conclusions:** This study provides a useful functional complement to physicochemical characterization and supports quality control and batch-to-batch consistency of RSV mRNA drug substances. In addition, the development strategy may also serve as a useful reference for the establishment of in vitro potency assays for other mRNA drug substances.

## 1. Introduction

Respiratory syncytial virus (RSV) remains a leading cause of severe lower respiratory tract infections globally, posing a significant threat to infants, young children, and the elderly [1,2]. Despite decades of research, the development of a safe and effective vaccine proved elusive until recent breakthroughs with both protein-based and mRNA-based platforms [3,4,5]. mRNA technology, validated by its monumental success during the COVID-19 pandemic, offers a highly adaptable and rapid pathway for vaccine development, enabling robust in vivo expression of antigenic proteins, such as the RSV prefusion F glycoprotein, which is critical for inducing neutralizing antibodies [6,7].

The manufacturing of mRNA drug substances (DS) is a complex process, and their critical quality attributes (CQAs), including integrity, purity, capping efficiency, and poly(A) tail length, are meticulously monitored using an orthogonal array of analytical techniques (e.g., capillary electrophoresis, liquid chromatography, and mass spectrometry) [8,9,10]. While these physicochemical methods are indispensable for ensuring product identity and purity, they serve only as indirect proxies for the vaccine’s primary biological function: the efficient translation into a correctly folded, immunogenic antigen. The ultimate in vivo potency of an RSV mRNA vaccine depends on this functional activity, which can be impaired by subtle alterations in the mRNA structure that are not detected by standard physicochemical assays [11,12,13]. This creates a critical gap in the control strategy, potentially allowing batches with suboptimal potency to pass quality control based solely on physicochemical and structural metrics.

Quality assessment of mRNA drug substances typically includes evaluation of physicochemical properties, sequence identity, cap structure and capping efficiency, poly(A) tail length, purity, impurities, biological activity, and safety. Among these, cell-based bioassays are the cornerstone for quantifying the biological activity, as they provide a holistic measure of functionality by capturing the entire process from cellular uptake to protein expression and presentation [14,15]. In general, a cell-based bioassay for mRNA drug substances involves complexing the mRNA with a transfection reagent, followed by in vitro transfection into mammalian cells to enable antigen expression. The expressed antigen can then be quantified using an appropriate detection method, such as a double-antibody sandwich ELISA. For an RSV mRNA drug substance, an ideal potency assay should quantitatively measure the expression of the encoded antigen (the prefusion F protein) and ideally reflect its immunogenic quality. The development of a robust, antigen-specific bioassay that is validated under Good Manufacturing Practice (GMP) principles is therefore essential for the quality control of mRNA products [16,17,18,19]. A key and often challenging attribute of such a potency assay is its discriminatory ability—the capacity to reliably detect differences in the biological activity among samples intentionally altered in their CQAs through process changes or degradation. However, there are currently no published reports on the systematic development of such an in vitro potency method for mRNA drug substances, nor on evaluating the differences in potency across samples with varying CQAs.

To address this unmet need in the burgeoning field of RSV mRNA vaccines, this study describes the development, validation, and application of a novel in vitro cell-based bioassay specifically designed to quantify the functional biological activity of an RSV mRNA drug substance encoding the prefusion F glycoprotein of RSV type A. The assay quantifies the expression of the membrane-bound RSV prefusion F glycoprotein in a susceptible, mechanism-related cell line using an optimized in vitro mRNA transfection method and a prefusion F protein-specific sandwich enzyme-linked immunosorbent assay (ELISA). To obtain a sufficiently sensitive, precise, accurate, and robust potency assay, parameters including the transfected cell line type and cell density, transfection reagent, transfection duration, and ELISA detection conditions were fully screened and optimized. Comprehensive validation was then performed in accordance with ICH Q2(R1) guidelines under GMP laboratory conditions to demonstrate assay specificity, accuracy, precision, linearity, and range [20]. More importantly, the assay’s discriminatory power was rigorously challenged by analyzing a panel of mRNA drug substance samples with broad variations in key CQAs, such as 5′ cap structure, capping efficiency, poly(A) tail length, and mRNA integrity/purity. These results provide a scientific basis for establishing specifications for key quality attributes of RSV mRNA drug substances. The successful application of this validated method to samples from different processes and to stability samples under forced degradation confirms its value as a sensitive and stability-indicating assay for the quality control of RSV mRNA drug substances. In the present study, RSV mRNA drug substance was used as a representative example to establish a systematic framework for cell-based potency assay development. By focusing on two key steps, namely transfection and antigen detection, this study provides a practical approach for evaluating mRNA potency. With appropriate optimization and validation, this general framework may also be informative for the development of potency assays for other mRNA drug substances or vaccines.

## 2. Materials and Methods

### 2.1. Materials

Cell lines *used* for mRNA transfection included the human liver carcinoma cell line HepG2 (American Type Culture Collection, ATCC, Manassas, VA, USA), human non-small cell lung carcinoma cell line A549 (ATCC), African green monkey kidney epithelial cell line Vero (ATCC), human epidermoid carcinoma type 2 cell line Hep-2 (ATCC), and human embryonic kidney cell line 293T (China Center for Type Culture Collection, CCTCC, Wuhan, China). To evaluate transfection efficiency, the following reagents were employed: Lipofectamine MessengerMAX (Thermo Fisher Scientific, Waltham, MA, USA), Lipofectamine 3000 (Thermo Fisher Scientific), Lipofectamine 2000 (Thermo Fisher Scientific), and jetMESSENGER (Polyplus, Illkirch, France). All transfections were performed according to the manufacturers’ protocols with minor modifications as required. For ELISA assays, the capture antibody specific to the prefusion F protein was obtained from Cambridge Bio, while the detection antibody, an HRP-conjugated monoclonal antibody against the RSV fusion protein, was purchased from Sinobiological. Dulbecco’s Modified Eagle Medium (DMEM) and 0.25% Trypsin-EDTA were sourced from Gibco, and Eagle’s Minimum Essential Medium (EMEM) was obtained from ATCC. Opti-MEM™ reduced-serum medium was acquired from Gibco, and fetal bovine serum (FBS) was purchased from HyClone (Logan, UT, USA). NP-40 lysis buffer and the Cell Counting Kit-8 (CCK-8) were provided by Beyotime Institute of Biotechnology. Single- and two-component TMB substrates were supplied by Sangon Biotech and Solarbio. All other reagents used were of biological or analytical grade.

### 2.2. Methods

#### 2.2.1. Preparation of RSV mRNA Drug Substance

mRNA drug substances encoding the RSV prefusion F protein were produced by In-norna, a biotechnology company based in China. An RSV linearized DNA plasmid template containing a T7 promoter sequence was used to initiate in vitro transcription by T7 RNA polymerase in the presence of nucleotide triphosphates (NTPs), thereby generating the primary RSV prefusion F protein mRNA strand. Following transcription, the DNA plasmid template was digested with DNase I. Tangential flow filtration was applied to exchange the buffer and remove residual NTPs. The mRNA was subsequently capped using a capping enzyme system (vaccinia capping enzyme and 2′-O-methyltransferase), which introduced a 5′ cap structure (Cap 0). This cap was further methylated to form the Cap 1 structure, enhancing stability and translational efficiency. The resulting capped mRNA underwent a multi-step purification process, including tangential flow filtration (molecular weight cutoff (MWCO) of 100 kDa, polyethersulfone (PES)), oligo dT chromatography (Sorbent: POROS™ Oligo (dT)25 Affinity Resin (Thermo Fisher Scientific, Waltham, MA, USA); buffer: 0.1 M NaCl), and bioburden-reducing filtration (0.2 μm polyether sulfone membrane filter),to remove residual DNA, enzymes, incomplete transcripts, and microorganisms. This yielded high-quality mRNA drug substances suitable for potency assay development and validation studies. One batch of mRNA drug substance used for the RSV vaccine Good Laboratory Practice (GLP) toxicology study was designated as the in-house reference standard for in vitro potency assay development, validation, and sample testing. Furthermore, mRNA drug substances with varying quality attributes—including poly(A) tail length, capping structure, capping efficiency, and integrity/purity—were generated and utilized for performance evaluation of the in vitro potency assay. mRNAs with different poly(A) tail lengths (60 nt, 95 nt, and 100 nt) were produced by modifying the poly(A) sequence in the DNA plasmid templates. Distinct capping structures (Cap 1, Cap 0, GCap, and uncapped (Ucap)) were obtained by adjusting the capping process. Additionally, a forced photodegradation study was performed under controlled conditions using cool white fluorescent lamps (4000 ± 500 lux) and near-UV lamps (0.09 mW/cm^2^) at a constant temperature of 25 °C. Samples were exposed for 0, 1, 2, 4, 7, and 14 days to generate mRNA drug substance samples with varying degrees of degradation, resulting in different levels of mRNA integrity and purity.

#### 2.2.2. Cell Culture and mRNA Transfection

HepG2, A549, Vero, Hep-2, and 293T cells were cultured in tissue culture-treated, barcoded T75 flasks (Corning, NY, USA) using the appropriate media supplemented with 10% fetal bovine serum (FBS). Vials were thawed in a 37 °C water bath, and upon thawing, cells were diluted 1:10, pelleted at 1000× *g* for 3 min, and resuspended in fresh culture medium after removal of the cryopreservation medium. The diluted cells were expanded and maintained in T75 flasks containing 30 mL of growth medium at various cell densities for 2–3 days (depending on the cell line) at 37 °C in 5% CO_2_, with medium changes and passaging performed according to cell density. Cells were harvested from T75 flasks in their respective culture media, with the FBS concentration consistently maintained at 10% for cell plating. Lipofectamine MessengerMAX or other mRNA transfection reagents were prepared by dilution into medium at ratios specified by the manufacturers’ protocols or optimized as required, followed by incubation at room temperature (RT) for 10 min. Reference standards and test samples were prediluted in Opti-MEM™ reduced-serum medium (Gibco, Thermo Fisher Scientific, Waltham, MA, USA) to a final mRNA concentration of 9.6 µg/mL. A two-fold serial dilution series was then prepared directly in the cell culture plate. The diluted mRNA was mixed 1:1 (*v*/*v*) with the prepared transfection reagent and incubated at RT for 5 min to allow formation of mRNA–transfection reagent complexes. Cells maintained under the above culture conditions were subsequently seeded into plates containing the complexes at a density of 1 × 10^5^ cells per well. Plates were incubated for 16–20 h at 37 °C to permit mRNA transfection and expression of the RSV prefusion F protein antigen.

#### 2.2.3. ELISA Detection for RSV Antigen Protein Expression

After completion of mRNA transfection, cell culture supernatants were removed, and 120 µL of NP-40 lysis buffer was added to each well. Cells were lysed by incubation for 10 min at 4 °C to release the expressed RSV prefusion F protein. The resulting lysates were transferred to microtiter plates pre-coated with a prefusion F protein specific antibody (Anti-F (RSV) CR9501; Cambridge Bio, Suzhou, China; Cat#01-07-0145) and incubated for 2 h at 37 °C. Following washing, 100 µL of HRP-conjugated anti-RSV-F antibody (Human respiratory syncytial virus (RSV) (A2) Fusion glycoprotein/RSV-F Antibody (HRP); Sino Biological Inc., Beijing, China; Cat#11049-R302-H) was added to each well and incubated for 1 h at 37 °C. After a subsequent wash, 100 µL of TMB substrate was added. The reaction was terminated after 10 min by addition of 50 µL of 2 M sulfuric acid, and absorbance was immediately measured at 450 nm (reference wavelength: 630 nm) using a microplate reader. Dose–response curves were generated by plotting the amount of transfected mRNA (x-axis) against the corrected absorbance (OD_450_–OD_630_; y-axis), which reflects the expression level of the RSV pre-fusion F protein. A four-parameter logistic (4PL) model was independently fitted for the reference and test samples. The relative in vitro potency was determined as the ratio of the EC_50_ of the reference standard to that of the test sample.

#### 2.2.4. Cell Viability Assay

Cell viability was assessed using the Cell Counting Kit-8 (CCK-8; Beyotime Institute of Biotechnology, Shanghai, China), a WST-8-based colorimetric assay. In this assay, WST-8 is reduced by dehydrogenases in viable cells to produce a water-soluble orange formazan dye, the amount of which is directly proportional to the number of living cells. Briefly, after mRNA transfection, the culture medium was replaced with fresh medium containing CCK-8 solution, and the cells were incubated at 37 °C for 1 h to allow for formazan formation. Cell viability was then determined by measuring absorbance at 450 nm, with 630 nm as the reference wavelength, using a microplate reader.

#### 2.2.5. Potency Assay Development

Comprehensive method development was conducted to obtain a sensitive, accurate, precise and robust in vitro potency assay. Five commercially available adherent cell lines with distinct endocytic capacities and innate immune signatures, including HepG2, A549, Vero, HEp-2, and 293T, were initially screened to compare mRNA transfection efficiency and RSV prefusion F protein expression. Cell densities ranging from 0.5 × 10^5^ to 2.0 × 10^5^ cells per well were evaluated to determine the optimal seeding density. To identify the most effective transfection reagent, four commercially available reagents, Lipofectamine MessengerMAX, Lipofectamine 3000, Lipofectamine 2000, and jetMESSENGER, were systematically assessed. In addition, the ratio of mRNA to transfection reagent was optimized to determine the appropriate reagent amount. Further optimization included a detailed evaluation of cell transfection duration and ELISA assay parameters for RSV prefusion F protein detection, such as coating and detection antibody concentrations, incubation time and temperature, and substrate solution type. Ultimately, these systematic evaluations resulted in the establishment of an optimized in vitro potency assay for the RSV mRNA drug substance.

#### 2.2.6. Potency Assay Validation

To confirm that this assay is suitable for the testing of RSV mRNA drug substances, an assay validation study in accordance with ICH Q2(R2) guidelines [20] was conducted in a quality control laboratory to determine the assay’s specificity, linearity, accuracy, precision and range, and robustness.

Specificity was assessed using negative controls and degraded samples to confirm selective detection of the biological activity of the target mRNA drug substance and to evaluate the stability-indicating capability of the assay. Accuracy was evaluated by comparing measured relative potency with expected potency across multiple predefined potency levels. Repeatability and intermediate precision were assessed to determine assay variability within a single run and across different days and analysts. Linearity was evaluated by analyzing the relationship between measured and expected relative potency over the predefined potency range, and the validated range was defined as the interval over which acceptable assay performance was demonstrated. Robustness was assessed by introducing deliberate variations in critical assay parameters and by evaluating the effect of cell passage number on assay performance.

A pre-defined acceptance criteria was established for all the validation items, shown in Table 1. In the validation, a GLP toxicology mRNA drug substance batch was used as in-house reference standard, and a similar but different batch of drug substance was used as testing sample.

#### 2.2.7. Discriminatory Performance of the Potency Assay on Varying Samples

After completing method development and validation, the performance of the assay was evaluated in distinguishing samples with varying potencies. These included samples with different poly(A) tail lengths (60–100 nt), cap structures (Cap1, Cap0, GCap, and Ucap), capping efficiencies (0–100%), and varying levels of mRNA integrity (42–98%) generated through light-exposure forced degradation. This application study provided a comprehensive assessment of the assay’s detection capability under practical conditions and further demonstrated the critical role of this in vitro potency assay in the quality control of RSV mRNA drug substances.

## 3. Results

### 3.1. Optimization of Cell Transfection Parameters

**Figure 1:**
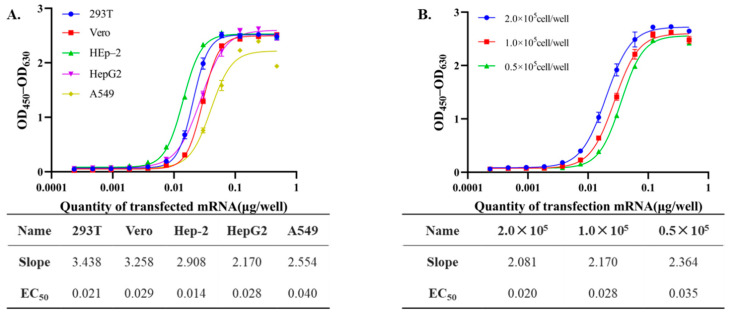
Optimization of cell-related conditions for the RSV mRNA potency assay. (**A**) Dose–response curves of RSV mRNA transfection in different cell lines (293T, Vero, HEp-2, HepG2, and A549). Cells were transfected with increasing amounts of RSV mRNA (0.0002–0.48 µg/well), and RSV prefusion F protein expression was measured by ELISA as OD_450_–OD_630_. The corresponding EC_50_ and slope values are summarized in the inset table. (**B**) Effect of HepG2 cell seeding density on RSV prefusion F protein expression. HepG2 cells were seeded at 0.5 × 10^5^, 1.0 × 10^5^, or 2.0 × 10^5^ cells/well and transfected with increasing amounts of RSV mRNA (0.0002–0.48 µg/well). Protein expression was measured by ELISA as OD_450_–OD_630_.

The choice of cell line type is an important determinant of the performance of in vitro potency assays for mRNA-based products, as different cell lines may vary in transfection efficiency, mRNA translation capacity, and protein expression profiles. Selecting a cell line with consistent responsiveness to mRNA transfection is critical for obtaining biologically meaningful and reproducible assay results [21]. Therefore, five commercially available adherent cell lines (293T, Vero, HEp-2, HepG2, and A549) were evaluated for their suitability in mRNA transfection and subsequent expression of the RSV prefusion F protein. These cell lines were selected across different cellular backgrounds, aiming to identify the most suitable cell line for the in vitro potency assay of mRNA drug substance. As shown in Figure 1A, it was observed that dose–response curves generated from RSV mRNA transfection revealed comparable transfection efficiency across 293T, Vero, HEp-2, and HepG2 cells, with well-defined sigmoidal profiles exhibiting distinct upper and lower asymptotes. Notably, A549 cells displayed a mild hook effect at higher mRNA concentrations, indicating potential saturation or non-linear response, which may compromise assay accuracy at high doses. Analysis of the linear dynamic range showed that both Hep-2 and HepG2 cells yielded three data points within the linear region, providing sufficient resolution for robust curve fitting and reliable EC_50_ determination. Among the tested cell lines, HepG2 exhibited a relatively flatter slope (2.170) than other cell lines (e.g., 293T: 3.438, Vero: 3.258), which may improve assay stability by reducing sensitivity to small variations in mRNA concentration. A less steep slope can be advantageous for minimizing variability in EC_50_ estimation and improving assay reproducibility and accuracy. In addition, the current USP guidance for mRNA vaccines also includes HepG2 cells as an in vitro model for potency evaluation [17]. Based on these considerations, HepG2 was selected as the cell line for subsequent assay optimization.

Cell density can markedly influence the performance of in vitro potency assays for mRNA drug substances, as excessively high or low cell density may affect transfection efficiency and protein expression levels. An appropriate cell density is therefore important to ensure efficient cellular uptake and a uniform response, thereby improving the accuracy and reproducibility of potency measurements. As shown in Figure 1B, HepG2 cells were seeded at three different densities (2.0 × 10^5^, 1.0 × 10^5^, and 0.5 × 10^5^ cells per well) in 96-well plates. All three densities generated clear sigmoidal dose–response curves with similar maximal responses and slopes. However, EC_50_ values increased consistently as cell density decreased, indicating reduced transfection efficiency at lower cell numbers. By contrast, the highest cell density (2.0 × 10^5^ cells/well) may result in cellular overcrowding and clustering, which could impair nutrient diffusion, medium access, and the uniformity of transfection, thereby affecting assay consistency and robustness. Therefore, a moderate cell density of 1.0 × 10^5^ cells per well was selected as the optimal condition for RSV mRNA transfection.

**Figure 2:**
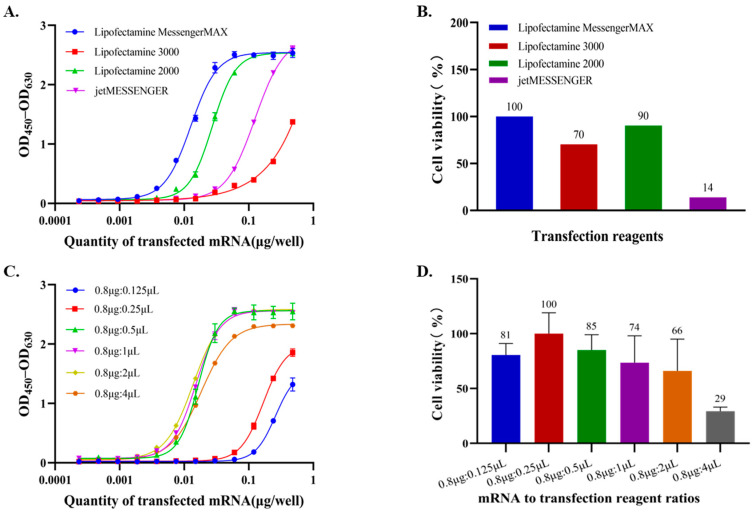
Optimization of transfection conditions for the RSV mRNA potency assay. (**A**) RSV prefusion F protein expression following transfection with different transfection reagents, measured by ELISA as OD_450_–OD_630_. (**B**) Cell viability after transfection with different transfection reagents, assessed using the Cell Counting Kit-8 (CCK-8) assay. (**C**) RSV prefusion F protein expression at different mRNA-to-transfection reagent ratios, measured by ELISA as OD_450_–OD_630_. (**D**) Cell viability at different mRNA-to-transfection reagent ratios, assessed using the CCK-8 assay.

The choice of transfection reagent can significantly influence the performance of an in vitro potency assay for mRNA drug substances, as it directly affects transfection efficiency, cellular uptake, and cytotoxicity. Different transfection reagents vary in their molecular structure and ability to deliver mRNA into cells, affecting protein expression levels and thus the accuracy and reproducibility of potency measurements. Four commercially available mRNA transfection reagents, including Lipofectamine MessengerMAX, Lipofectamine 2000, Lipofectamine 3000 (all from Thermo Fisher Scientific), and jetMESSENGER (Polyplus), were evaluated for their suitability in this potency assay. As shown in Figure 2A, ELISA analysis of antigen expression demonstrated that both Lipofectamine MessengerMAX and Lipofectamine 2000 generated sigmoidal dose–response curves with clear upper asymptotes, indicating robust and saturable transfection performance.

In contrast, Lipofectamine 3000 and jetMESSENGER failed to reach a plateau, suggesting incomplete or less efficient transfection at higher mRNA doses. The EC_50_ values followed the order: Lipofectamine MessengerMAX < Lipofectamine 2000 < jetMESSENGER < Lipofectamine 3000, indicating that Lipofectamine MessengerMAX showed the highest transfection efficiency among the reagents tested. Cytotoxicity was assessed using the CCK-8 assay. As shown in Figure 2B, Lipofectamine MessengerMAX resulted in the highest cell viability among the tested reagents at an mRNA dose of 0.48 µg/well. Specifically, cell viability was approximately 100% with Lipofectamine MessengerMAX, compared with approximately 90% for Lipofectamine 2000, 70% for Lipofectamine 3000, and 14% for jetMESSENGER. These results indicate that Lipofectamine MessengerMAX not only provides high transfection efficiency but also exhibits minimal cytotoxicity.

It has been reported that the ratio of nucleic acid to transfection reagent, often discussed in the context of N/P ratio, can significantly influence cellular transfection efficiency [22,23]. Therefore, to further optimize the transfection conditions, we investigated the effects of the mRNA-to-transfection reagent ratio on both transfection efficiency and cell viability. As shown in Figure 2C, at ratios ranging from 0.8 μg mRNA: 0.5 μL to 0.8 μg:4 μL, sigmoidal dose–response curves were observed with defined lower and upper asymptotes, and no significant differences in EC_50_ or maximum expression levels. However, when the amount of transfection reagent was reduced below the ratio of 0.8 μg:0.5 μL (e.g., 0.8 μg:0.125 μL or 0.8 μg:0.25 μL), transfection efficiency declined markedly, and the dose–response curves failed to reach an upper plateau, indicating insufficient complex formation and intracellular delivery. Cell viability remained high across ratios ranging from 0.8 μg:0.125 μL to 0.8 μg:1 μL (Figure 2D). In contrast, when the amount of transfection reagent exceeded 0.8 μg:1 μL, cytotoxicity became evident, with cell viability decreasing to approximately 66% at 0.8 μg:2 μL and 29% at 0.8 μg:4 μL. These results indicate that increasing the amount of transfection reagent improved transfection efficiency only up to a certain point, whereas excessive reagent levels caused substantial cytotoxicity and could compromise assay reliability. Therefore, Lipofectamine MessengerMAX and a moderate mRNA-to-transfection reagent ratio of 0.8 μg:1 μL were selected as the final transfection conditions.

**Figure 3:**
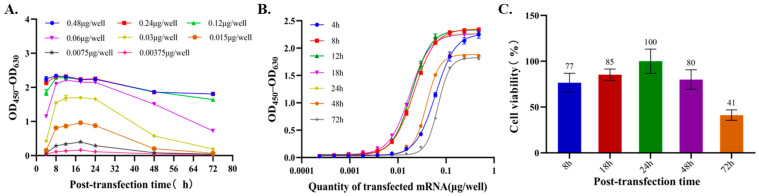
Transfection kinetics and cytotoxicity assessment for the RSV mRNA potency assay. (**A**) Time-dependent expression kinetics of RSV prefusion F protein at different transfected mRNA doses (0.00375–0.48 µg/well), measured by ELISA as OD_450_–OD_630_. (**B**) Sigmoidal dose–response curves of RSV prefusion F protein expression at different time points after transfection (4, 8, 12, 18, 24, 48, and 72 h). (**C**) Cell viability at different time points after transfection (8–72 h) with 0.48 µg/well RSV mRNA, assessed using the CCK-8 assay.

The expression kinetics of RSV prefusion F protein and cell viability were markedly influenced by transfection duration, reflecting the dynamic relationship between antigen production and cell health. As shown in Figure 3A, RSV prefusion F protein expression, quantified as OD_450_–OD_630_, exhibited a clear dose- and time-dependent pattern. At the early time point of 4 h post-transfection, low levels of RSV prefusion F protein expression were detectable across all mRNA doses, indicating the initiation of translation. Expression increased rapidly between 8 and 12 h and reached a plateau between 12 and 24 h, with the highest signals observed at mRNA doses of 0.12–0.48 µg/well. Thereafter, expression gradually declined at 48 and 72 h, suggesting possible degradation of the expressed protein or attenuation of translation over time. This expression profile was also dependent on the amount of transfected mRNA (Figure 3A). Higher mRNA inputs (e.g., 0.12–0.48 µg/well) resulted in earlier and stronger expression peaks, whereas lower doses showed a delayed onset and reduced peak expression. However, prolonged transfection times beyond 24 h consistently resulted in diminished expression across all tested doses, indicating a temporal limitation of mRNA-driven protein production. As shown in Figure 3B, sigmoidal dose–response curves were observed at all evaluated time points from 4 to 72 h post-transfection. However, higher EC_50_ values and poorer curve shapes were observed at excessively short (4 h) or prolonged (48 and 72 h) transfection times, suggesting that assay performance may be suboptimal under these conditions. Based on these results, a transfection time of 8–24 h was considered appropriate for the in vitro potency assay. In parallel, cell viability was assessed using the CCK-8 assay at an mRNA dose of 0.48 μg/well (Figure 3C) and showed a distinct time-dependent trend. Cell viability peaked at 24 h post-transfection (approximately 100%), indicating an optimal cellular state during the early-to-mid phase of protein expression. Thereafter, viability gradually declined to approximately 80% at 48 h and further decreased to 41% at 72 h. This decline temporally coincided with the reduction in RSV prefusion F protein expression, suggesting that extended transfection duration may induce cytotoxic effects. This may be associated with persistent intracellular mRNA, innate immune activation, or metabolic stress, thereby compromising both cell health and sustained protein production.

Collectively, these results indicate that RSV prefusion F protein expression was optimal between 8 and 24 h post-transfection, providing a balance between high antigen expression and acceptable cell viability. Because both protein expression and cell viability declined beyond this period, transfection duration was identified as a critical parameter influencing assay performance. Based on these findings, a transfection time of 16–24 h was selected as the final assay condition to enhance robustness and operational flexibility during routine sample testing.

### 3.2. Optimization of ELISA Detection Parameters for RSV Prefusion F Protein Quantification

A checkerboard titration was performed to optimize the concentrations of the capture and detection antibodies used in the sandwich ELISA, and the results are summarized in Table 2. The signal window was defined as the ratio of the OD_450_–OD_630_ values obtained at high (0.48 μg/well) and low (0.0002 μg/well) mRNA concentrations. A recombinant human monoclonal antibody specific to the RSV prefusion F protein, Anti-F (RSV) CR9501, was used as the capture antibody, and HRP-conjugated anti-RSV-F antibody was used as the detection antibody. As shown in Table 2, increasing the capture antibody concentration from 0.25 μg/mL to 2 μg/mL resulted in an increase in signal window, with the maximum value observed at 1 μg/mL. Therefore, 1 μg/mL was selected as the optimal capture antibody concentration. For the detection antibody, lower concentrations were associated with reduced OD signals. Although both 0.5 μg/mL and 0.2 μg/mL produced signal windows greater than 2.0, the signal declined more sharply when the concentration was reduced below 0.2 μg/mL, indicating reduced sensitivity and dynamic range. Therefore, 0.3 μg/mL was selected as the optimal detection antibody concentration to balance sensitivity, signal stability, and reproducibility.

**Figure 4:**
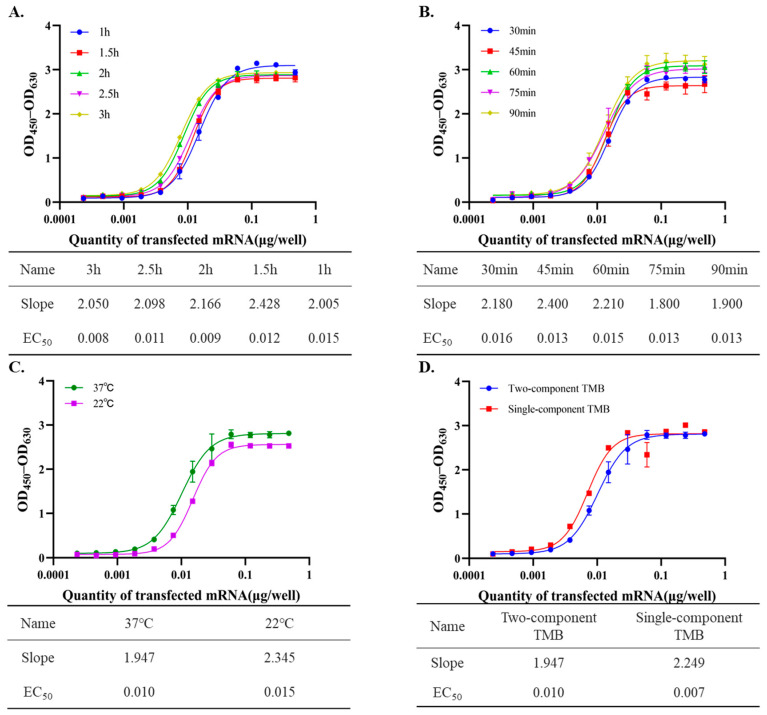
Optimization of ELISA detection conditions for RSV prefusion F protein quantification. (**A**) Determination of the optimal incubation time between cell lysate and the capture antibody. Serial dilutions of lysates from mRNA-transfected cells were incubated with the capture antibody for different durations. (**B**) Determination of the optimal incubation time for the detection antibody with the captured antigen. (**C**) Effect of incubation temperature on the binding of the capture or detection antibody to the antigen in the ELISA assay. (**D**) Comparison of color development using single-component and two-component TMB substrates.

In addition, the incubation time of the cell lysate with the capture antibody was optimized. As shown in Figure 4A, incubation for 1.5 to 3 h generated consistent sigmoidal dose–response curves. Although a 1 h incubation also generated an acceptable curve, the EC_50_ was slightly higher (0.015 μg versus approximately 0.008–0.011 μg), suggesting less efficient antigen capture under this condition. Therefore, an incubation time of 1.5 to 3 h was considered optimal, as it provided efficient antigen capture without affecting assay performance.

As illustrated in Figure 4B, incubation of the detection antibody with the captured antigen for 30–90 min yielded highly consistent sigmoidal dose–response profiles, with no significant differences observed among the tested time points. All time points within this range provided reliable signal output and sensitivity, indicating that 30–90 min is an acceptable and flexible incubation window for detection antibody binding to the captured antigen.

The effect of incubation temperature on the binding of capture and detection antibodies to the antigen in the ELISA was evaluated at 37 °C and room temperature (22 °C). As shown in Figure 4C, dose–response curves at both temperatures exhibited sigmoidal responses with increasing amounts of transfected mRNA. However, the curve obtained at 37 °C showed a shallower slope (1.947) than that obtained at 22 °C (2.345), suggesting a more moderate response across the detection range, which may improve the assay’s ability to discriminate between samples. In addition, the EC_50_ value at 37 °C was lower than that at room temperature, indicating improved assay sensitivity at the higher temperature. Based on these results, 37 °C was selected as the optimal incubation temperature for antibody–antigen binding, as it provided better sensitivity and a more robust signal response across the tested mRNA concentrations.

To evaluate substrate performance, single-component and two-component TMB substrates from Sangon Biotech and Solarbio were compared. As shown in Figure 4D, all substrates generated valid sigmoidal dose–response curves. However, the single-component substrate showed high initial sensitivity and steeper slopes, but its signal was unstable, with precipitate formation and signal decay occurring within 20 min after the reaction was stopped, which limited its practical utility. In contrast, the two-component substrates showed better stability and sustained signal over time. Among them, the two-component TMB substrate from Sangon Biotech produced the highest signal intensity and the best overall performance, and was therefore selected for subsequent use in the assay.

### 3.3. Comprehensive Validation Result of Optimized Bioassay

We performed a comprehensive validation of the assay in accordance with ICH Q2 guidelines to demonstrate its specificity, accuracy, precision, linearity, range, and robustness [20].

**Figure 5:**
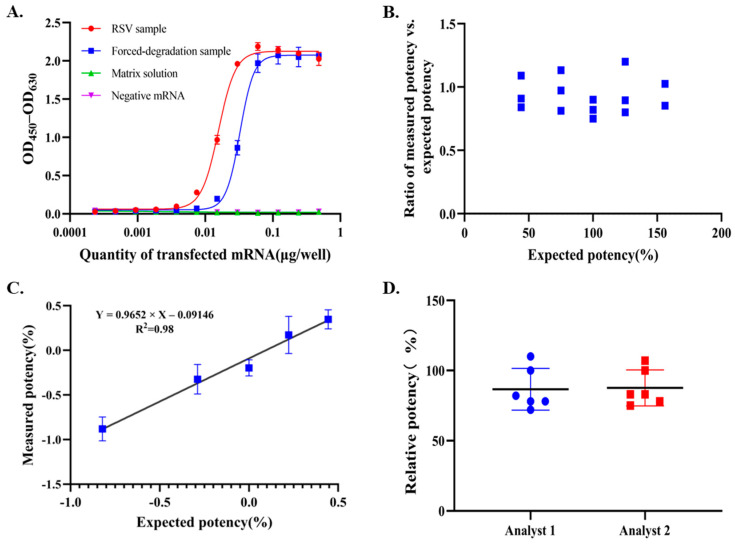
Validation of assay specificity, accuracy, linearity, and precision. (**A**) Dose–response curves of the matrix solution, unrelated mRNA drug substance (Herpes zoster mRNA), and native and degraded RSV mRNA drug substance samples. (**B**) Accuracy of the assay, shown as the ratio of measured to expected values. Each dot represents one measurement. (**C**) Linearity of the assay. Each point represents the mean of three replicates. (**D**) Precision of the assay, shown as the agreement among replicate measurements obtained by the same analyst and by two analysts on different days.

Specificity is the ability to measure the analyte of interest accurately and specifically. The specificity of the method was evaluated using the matrix solution and an unrelated mRNA drug substance (Herpes zoster mRNA) as negative controls. As shown in Figure 5A, neither control produced a dose–response curve, whereas the RSV mRNA drug substance generated a clear sigmoidal response, indicating that the assay was specific for RSV mRNA. To further assess the stability-indicating capability of the method, native and degraded RSV mRNA drug substance samples were compared. The degraded samples showed a clear shift in the dose–response curve and a significant increase in EC_50_, indicating a substantial loss of biological activity (Figure 5A).

Accuracy was assessed by comparing measured potency with theoretical potency using three independent preparations tested at five levels across the reportable range (44–156%). The relative bias (RB) across the five potency levels ranged from −25% to 13%, which was within the predefined acceptance criterion of ±50%. Agreement between measured and expected potency values was further evaluated using the measured-to-expected ratio. As shown in Figure 5B, all measured-to-expected ratios were within 0.8–1.2 and were generally distributed around 1.0, demonstrating acceptable accuracy. The assay also showed good linearity, with an R^2^ of 0.98 and a slope of 0.97 in the linear regression analysis (Figure 5C).

Precision was evaluated as repeatability and intermediate precision. Six replicate analyses performed on the same day yielded an intra-assay CV of 18%, whereas twelve independent assays performed by two analysts on different days yielded an inter-assay CV of 16%, indicating acceptable assay precision (Figure 5D).

The robustness of an analytical procedure is a measure of capacity to meet the expected performance criteria during normal use. Robustness is tested by deliberate variations in analytical procedure parameters. Since the physiological state and viability of cells vary across different passages, which may subsequently impact mRNA transfection efficiency, we evaluated the transfection efficiency of the RSV mRNA drug substance across a broad range of cell passages (passage 6 to passage 35). The results demonstrated no significant impact on transfection efficiency throughout passages 6 to passage 35, as indicated by robust and consistent sigmoidal dose–response curves with comparable EC_50_ and slope values. Therefore, cells within this passage range are suitable for transfection. During method development, sigmoidal dose–response curves remained consistent despite deliberate variations in several critical assay parameters, including transfection time (16–24 h), cell lysate incubation time with the capture antibody (1.5–3 h), and secondary antibody incubation time (30–90 min). These results further support the ruggedness of the assay and its suitability for routine quality control testing.

Overall, the method met the predefined validation criteria for specificity, accuracy, precision, linearity and range, robustness in accordance with ICH Q2, supporting its validity and routine applicability under the conditions evaluated in this study.

### 3.4. Assay Sensitivity to Key CQAs

For mRNA drug substances, critical quality attributes (CQAs), such as cap structure, capping efficiency, poly(A) tail length, and mRNA integrity, may affect protein expression efficiency and ultimately influence product efficacy. To evaluate the discriminatory capability of the assay, mRNA drug substance samples covering a broad range of variations in these key CQAs, including 5′ cap structure, capping efficiency, shortened poly(A) tails, and mRNA integrity, were tested using the established potency assay under representative testing conditions.

**Figure 6:**
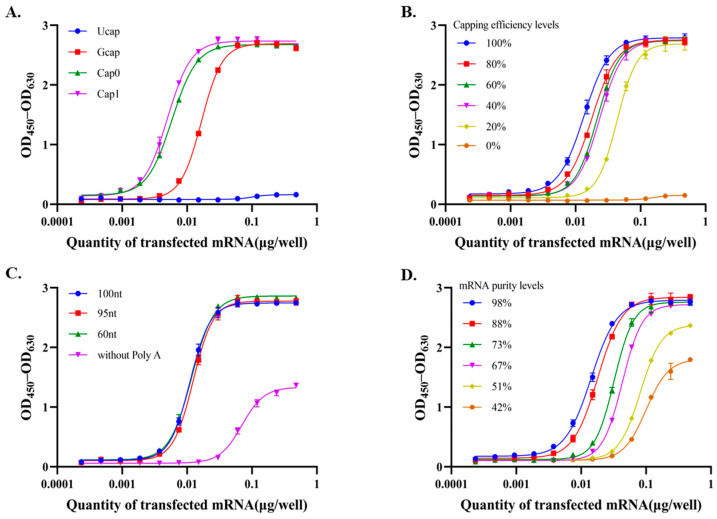
Discriminatory power of the assay for samples with different critical quality attributes. (**A**) Potency for mRNA with different cap structures. (**B**) Potency for mRNA with different capping efficiency. (**C**) Potency for mRNA with different lengths of poly(A) tails. (**D**) Potency for mRNA with different mRNA integrity.

The 5′ cap structure of mRNA can occur in several forms, including Cap1, Cap0, GCap, and UCap, each of which may differentially affect mRNA translation efficiency. Cap0 consists of a 7-methylguanosine (m7G) linked to the first nucleotide, whereas Cap1 contains an additional 2′-O-methylation on the ribose of the first nucleotide. GCap refers to the initial addition of a guanine cap before methylation, while uncapped mRNA (UCap) lacks these protective modifications. Using the optimized potency assay, we assessed the effect of these cap structures on RSV mRNA translation efficiency. As shown in Figure 6A and Table 3, the results demonstrated that UCap mRNA did not produce a measurable dose–response curve, whereas GCap mRNA showed only 28% relative biological activity, markedly lower than that of Cap0 (81%) and Cap1 (95%). The 5′ cap is a protective structure that safeguards RNA from exonuclease cleavage in vivo, regulates pre-mRNA splicing, and initiates mRNA translation as well as nuclear export. Furthermore, 2 -O-methylation of the 5 cap structure is a highly desirable property for increasing and enhancing the protein production from the mRNA after its transcription [8,9]. Accordingly, the assay clearly discriminated against mRNAs with different cap structures, with biological activity ranked as Cap1 > Cap0 > GCap > Ucap.

The proportion of Cap1 (capping efficiency) is a critical metric for evaluating batch-to-batch consistency during the production of mRNA drug substances. Consequently, we further investigate the impact of capping efficiency on the translation of RSV mRNA. We formulated samples with Cap1 ratios ranging from 0% to 100% by mixing defined amounts of Cap1 and Ucap mRNA. These samples were subsequently evaluated using the optimized assay. As illustrated in Figure 6B and Table 3, the results demonstrated a direct correlation between capping efficiency and potency, which is attributed to the critical role of the Cap1 structure in supporting efficient translation and mRNA stability. Furthermore, the observed positive correlation validates the method’s ability to accurately discriminate between samples with varying capping efficiencies.

The 3′ poly(A) tail plays a critical role in mRNA translation by protecting the transcript against degradation, enhancing stability, and improving translational efficiency. It functions synergistically with the 5′ m7G cap structure to promote mRNA circularization, thereby facilitating translation initiation [11,12]. As shown in Figure 6C and Table 3, analysis of poly(A) tail length variants using the optimized potency assay revealed that tailless mRNA exhibited a significantly reduced response, retaining only 16% relative biological activity. In contrast, mRNAs with poly(A) tails of at least 60 nt demonstrated comparable potencies (98%, 85%, and 91%), with no significant differences observed among them. These findings align with published studies demonstrating that highly translated transcripts can possess short poly(A) tails of approximately 30 adenosines [24], a length sufficient to accommodate a single cytoplasmic poly(A)-binding protein (PABPC) molecule to promote efficient translation. Collectively, these data confirm that the optimized potency assay can effectively discriminate the biological activity of mRNAs based on their poly(A) tail lengths.

mRNA integrity directly determines the quantity and quality of intact translation templates, thereby impacting protein expression efficiency. To investigate this correlation, we prepared samples with varying levels of degradation through light exposure for different durations. As shown in Figure 6D and Table 3, mRNA with higher integrity demonstrated correspondingly higher potency. This positive correlation confirms that potency is directly proportional to the amount of intact mRNA present in the samples. Furthermore, these results reaffirm the assay’s robust sensitivity.

Collectively, the data presented above demonstrates the excellent discriminatory power of our cell-based assay in evaluating samples with varying levels of these CQAs. Given the complexity of mRNA manufacturing and molecular structure, there may exist additional, as yet unrecognized attributes that impact protein expression, which are not detectable by conventional physicochemical methods. This highly discriminative in vitro potency assay is capable of identifying such latent product quality risks, thereby ensuring quality control and batch-to-batch consistency of mRNA drug substances.

## 4. Discussion

A cell-based in vitro relative potency assay was successfully developed and validated to assess the functional activity of RSV mRNA vaccine drug substances. Through systematic optimization of critical parameters, including the cell line (HepG2), transfection reagent (Lipofectamine MessengerMAX), cell density, mRNA-to-reagent ratio, transfection duration, and ELISA detection conditions, a robust, accurate, and highly reproducible bioassay was established.

The selection of appropriate cell lines and transfection reagents is a critical determinant in developing a robust and biologically relevant in vitro relative potency assay for mRNA-based products. HepG2 cells were selected in this study because they exhibit high transfection efficiency and good reproducibility in lipid nanoparticle-mediated delivery and have been widely used as a model for in vitro transfection evaluations [17,21,25]. This makes them suitable for detecting changes in intrinsic mRNA quality attributes that affect translation and protein expression at the Drug Substance (DS) stage. However, the HepG2-based assay is not intended to directly predict the magnitude of in vivo immunogenicity, as HepG2 cells do not fully reflect the physiological context of intramuscular vaccination, where immune cells such as dendritic cells are involved in antigen uptake, processing, presentation, and immune activation. Therefore, the main role of this assay is to detect changes in intrinsic mRNA quality under controlled in vitro conditions. Product-specific acceptance criteria will need to be established through bridging studies that correlate in vitro DS potency data with in vivo immunogenicity data from the corresponding Drug Product (DP) across multiple representative batches. Despite its acknowledged limitations and biological differences from immune cells, HepG2 cells remain a reliable tool for rapid in vitro assessment of mRNA transfection and expression efficiency, providing robust support for preliminary screening and batch-to-batch consistency evaluation at the DS stage. Similarly, Lipofectamine MessengerMAX was chosen as the transfection reagent based on its superior ability to deliver modified mRNA into mammalian cells while maintaining low cytotoxicity, as supported by the dynamic transfection and cell viability studies. Systematic comparisons with alternative reagents revealed significant advantages in both expression levels and cytotoxicity when using MessengerMAX, underscoring the importance of optimizing transfection reagent selection. These findings highlight that a thorough and rational evaluation of both cellular and delivery components is essential for establishing a functionally meaningful in vitro potency method.

For broader applicability across different mRNA drug substances, this approach provides a foundational framework. The principles of cell line suitability, such as translational competence and low variability, and reagent compatibility, such as high efficiency and low toxicity, may be generalized, although specific parameters may require adjustment for different mRNA constructs or targets. Therefore, the detailed optimization of cell type and transfection conditions described here not only strengthens the validity of the current RSV mRNA vaccine drug substance potency assay but also provides a practical strategy for the development of standardized and transferable in vitro potency assays for a broad range of mRNA vaccines and therapeutics. Collectively, this work paves the way toward more predictive, mechanism-based quality control methods that capture the biological activity of mRNA products beyond mere physicochemical quantification.

Validation according to ICH Q2 guidelines confirmed that the assay exhibits excellent specificity, linearity, accuracy, and precision. Moreover, its ability to discriminate variations in critical quality attributes (CQAs), such as reduced capping efficiency or shortened poly(A) tails, directly reflects the effects of these molecular attributes on mRNA translational efficiency and stability in a cellular context [26,27]. In previous studies, capping efficiency, poly(A) tail length, and mRNA modification variants were evaluated using liquid chromatography/mass spectrometry [28]. In contrast, the assay established in the present study is designed to measure mRNA expression efficiency in a biologically relevant cell-based model. This design enables not only the accurate discrimination of CQA-related variations, but also the direct correlation of molecular attributes with intracellular translation efficiency and stability. Accordingly, this approach overcomes the limitations of conventional structural analyses and enables a comprehensive evaluation of mRNA drug substance quality [26]. The assay serves not merely as an in vitro analytical method for assessing mRNA expression efficiency, but also as an integrated platform for the comprehensive quality evaluation of the drug substance.

A limitation of the current assay is that it measures folded pre-F protein in cell lysates and therefore does not directly assess whether the encoded antigen is presented to the immune system through cell-surface expression or secretion. These downstream events are important for vaccine function in vivo and may be more directly evaluated by methods such as cell-based ELISA or flow cytometry. However, the purpose of the present assay was to assess potency at the Drug Substance (DS) stage, where the main focus is the intrinsic functionality of the mRNA, including its ability to support translation and correct folding of the encoded antigen. These properties are critical quality attributes for DS characterization and are directly relevant to process development and quality control. By comparison, antigen presentation and immunogenicity are influenced not only by the mRNA itself, but also by formulation-related and biological factors, such as LNP-mediated delivery, cellular uptake, intracellular processing, biodistribution, and host immune responses. Therefore, these aspects are more appropriately evaluated at the Drug Product (DP) stage and in vivo. In this context, the current lysate-based assay provides an upstream functional readout for DS evaluation and should be considered complementary to DP-stage assays and in vivo immunogenicity studies.

## 5. Conclusions

In conclusion, this study successfully established a cell-based in vitro potency assay for the RSV mRNA vaccine drug substance encoding the prefusion (pre-F) protein of RSV type A, an antigen widely recognized as an important target in RSV vaccine development because of its epidemiological relevance and strong immunogenicity.

The assay was validated to demonstrate specificity, accuracy, precision, and strong discriminatory power with respect to critical mRNA quality attributes. This method effectively bridges the gap between physicochemical characterization and biological activity, serving as a key tool for quality control and batch-to-batch consistency of RSV mRNA drug substances. Moreover, the potency assay functions not only as a pivotal functional test for RSV mRNA vaccine drug substances but also as a reference framework for the development of similar in vitro potency assays applicable to other mRNA-based vaccines and therapeutics. Although the general technical framework of the present assay may provide a useful reference for other mRNA drug substances, its direct application beyond RSV mRNA should be interpreted with caution. mRNA constructions may differ substantially in structural complexity, 5′-UTR/3′-UTR architecture, codon usage, and expression characteristics, all of which may influence protein expression and assay performance. As a result, key assay parameters, including host cell line selection, transfection reagent choice, transfection conditions, and the timing of expression analysis, may require case-specific optimization for each individual mRNA construct. Therefore, the current assay should be considered a methodological framework that may serve as a useful starting point for other mRNA-based products, rather than a universally transferable method without further optimization and validation.

## Figures and Tables

**Table 1:** Validation parameters and acceptance criteria of the potency assay.

Validation Parameters	Acceptance Criteria
Specificity	The Matrix solution and negative control solution (Unrelated sample solutions) should show no dose response. RSV sample solutions should show a significant dose response with defined lower and upper asymptotes. Biological activity of the forced degradation sample at elevated temperature should be decreased compared to that of the sample solution.
Accuracy	Relative bias at each potency level (44%, 75%, 100%, 125%, 156%) should be within ±50%, and a trend was not observed across potency levels.
Precision	Repeatability: Six independent sample solutions are tested by one analyst on one day, and the RSD should be no more than 30%.
	Intermediate Precision: Six independent sample solutions are tested by another analyst on another day. The RSD of the six independent sample solutions should be no more than 30%, and the RSD of the twelve independent sample solutions by the two analysts should be no more than 30%.
Linearity and Range	A linear relationship should be observed for Measured vs. Expected Potency with R^2^ no less than 0.95 and a slope within 0.8~1.2.
Robustness	Deliberate variation in cell passage number

**Table 2:** Optimization of capture and detection antibody concentrations by checkerboard titration in the sandwich ELISA.

Detection Antibody										
Captured antibody	Concentration	0.5 μg/mL		0.2 μg/mL		0.1 μg/mL		0.067 μg/mL		Transfected mRNA (μg/well)
		OD_450_–OD_630_	Signal window	OD_450_–OD_630_	Signal window	OD_450_–OD_630_	Signal window	OD_450_–OD_630_	Signal window	
	2 μg/mL	2.558	2.500	2.151	2.118	1.024	1.014	1.550	1.532	0.48
		0.058		0.033		0.010		0.018		0.0002
	1 μg/mL	2.551	2.506	2.126	2.097	1.022	1.010	1.351	1.334	0.48
		0.045		0.029		0.012		0.017		0.0002
	0.5 μg/mL	2.275	2.245	1.896	1.870	1.006	0.996	1.071	1.058	0.48
		0.030		0.026		0.010		0.013		0.0002
	0.25 μg/mL	1.792	1.796	1.186	1.193	0.648	0.648	0.557	0.555	0.48
		−0.004		−0.007		0.000		0.002		0.0002

Note: The signal window was defined as the ratio of the OD_450_–OD_630_ values obtained at high (0.48 μg/well) and low (0.0002 μg/well) mRNA concentrations. Anti-F (RSV) CR9501 was used as the capture antibody, and HRP-conjugated anti-RSV-F antibody was used as the detection antibody.

**Table 3:** Potency Across samples with varying levels of CQAs.

Critical Quality Attribute		Potency	Critical Quality Attribute		Potency
Cap structure	Ucap	5%	Poly(A) tail length	100 nt	98%
	Gcap	28%		95 nt	85%
	Cap0	81%		60 nt	91%
	Cap1	95%		without poly(A) tail	16%
Capping efficiency	100%	100%	Purity	98%	93%
	80%	78%		88%	67%
	60%	64%		73%	38%
	40%	58%		67%	28%
	20%	32%		51%	16%
	0%	9%		42%	13%

## Data Availability

The original contributions presented in this study are included in the article. Further inquiries can be directed to the corresponding authors.
